# A spiking network model for clustering report in a visual working memory task

**DOI:** 10.3389/fncom.2022.1030073

**Published:** 2023-01-12

**Authors:** Lixing Lei, Mengya Zhang, Tingyu Li, Yelin Dong, Da-Hui Wang

**Affiliations:** ^1^School of Systems Science, Beijing Normal University, Beijing, China; ^2^Department of Brain and Cognitive Sciences, Center for Visual Science, University of Rochester, Rochester, NY, United States; ^3^State Key Laboratory of Cognitive Neuroscience and Learning, Beijing Normal University, Beijing, China; ^4^Beijing Key Laboratory of Brain Imaging and Connectomics, Beijing Normal University, Beijing, China

**Keywords:** working memory, clustering report, heterogeneity, STP, spiking network

## Abstract

**Introduction:**

Working memory (WM) plays a key role in many cognitive processes, and great interest has been attracted by WM for many decades. Recently, it has been observed that the reports of the memorized color sampled from a uniform distribution are clustered, and the report error for the stimulus follows a Gaussian distribution.

**Methods:**

Based on the well-established ring model for visuospatial WM, we constructed a spiking network model with heterogeneous connectivity and embedded short-term plasticity (STP) to investigate the neurodynamic mechanisms behind this interesting phenomenon.

**Results:**

As a result, our model reproduced the clustering report given stimuli sampled from a uniform distribution and the error of the report following a Gaussian distribution. Perturbation studies showed that the heterogeneity of connectivity and STP are necessary to explain experimental observations.

**Conclusion:**

Our model provides a new perspective on the phenomenon of visual WM in experiments.

## Introduction

Working memory (WM), the ability to maintain and manipulate information internally, is critical for cognition and executive control of behavior (Alan, 1992). Thus, the precision of information in WM is important for subsequent cognition and behavior. However, precision fades, and errors arise in WM over time (Shintaro et al., 1989; Weiwei and Luck, 2009; Hyeyoung et al., 2017; Rosanne et al., 2019). Errors in WM are thought to originate from random noise, which causes neural representation to diffuse away from the initial state. The continuous attractor models (Albert et al., 2000; Klaus et al., 2014; Sebastian and Bays, 2018) have shown the drift of memorized information caused by noise in WM. Recently, Panichello et al. (2019) reported an interesting phenomenon in a color delayed-estimation task, where the participants were requested to remember the stimulus’s color that was uniformly sampled from a color wheel and presented to them in a brief period and then they reported the cued color stored in WM after various delay periods. The authors found that the reports were clustered and that the report error followed a Gaussian distribution. They used a stochastic differential equation (SDE) to explain the memory error using the diffusion driven by noise in the neural representation and the drift toward discrete attractor states. They further transformed the SDE into the Fokker–Planck equation (FPE) and obtained the evolution of the probability density function of the memory trace, which fits well with the experimental data. However, the neural mechanism underlying this phenomenon requires further investigation.

In this study, we constructed a spiking network model to explore the neural mechanism underlying the clustering report and Gaussian distribution of report error. The model follows the tradition of the ring model for WM, which was originally proposed to investigate spatial WM (Albert et al., 2000) and was then applied to explore the mechanism underlying the WM capacity limit (Wei et al., 2012) and other characteristics of WM (Fredrik et al., 2009). We introduced the heterogeneity to the connectivity of the ring model to generate “discrete-like” attractor state and the short-term plasticity (STP) to control the drift of the memory trace (Gianluigi et al., 2008; Alan et al., 2012; Alexander et al., 2019). Our model successfully reproduced the clustering report and Gaussian distribution of report error, which are consistent with experimental observations (Panichello et al., 2019).

## Materials and methods

### Neuronal dynamics

The neurons are modeled as leaky integrate and fire neurons (Henry, 1988), and the subthreshold membrane potential *V*(*t*) obeys the dynamics as follows (Alan, 1992):


(1) $$C_{\text{m}}\,\frac{d\,V\,\left(t\right)}{d\,t}=-g_{\text{L}}\,\left(V\,\left(t\right)-V_{\text{L}}\right)+I_{\text{syn}}\,\left(t\right)$$

where *C*_*m*_ is the membrane capacitance, *g*_*L*_ is the leaky conductance, *V*_*L*_ is the resting potential, and *I*_syn_(*t*) is the total synaptic current input to a neuron. When the membrane potential *V*(*t*) exceeds the threshold potential *V*_*th*_, the model neuron fires a spike, and the membrane potential *V*(*t*) is reset to *V*_*res*_. During the refractory period for τ ms after a spike, the membrane potential is maintained as *V*_*res*_. The parameters are set as follows: *C*_m_ = 0.5*nF*, *g*_*L*_ = 0.025μ*S*, and τ = 2*ms* for pyramidal cell (or E neuron); *C*_m_ = 0.2*nF*, *g*_*L*_ = 0.020μ*S*, and τ = 1*ms* for inhibitory interneuron (or I neuron); and *V*_*L*_ = -70*mV*, *V*_*th*_ = -50*mV*, and *V*_*res*_ = -60*mV* for both E and I neurons following literature (Todd and Miller, 1997; Wang, 1999).

### Synaptic dynamics

The synaptic current includes the recurrent synaptic currents, task-irrelevant background noise, and task-relevant inputs. The recurrent currents *I*_NMDA_ and *I*_GABA_ are mediated by the *N*-methyl-D-aspartic acid receptor (NMDAR) and γ Aminobutyric Acid receptor (GABAR), respectively. Considering that NMDAR mediated current plays a key role in the persistent activity of network during the delay period (Wang, 1999), we omitted the fast excitatory recurrent current mediated by α-Amino-3-hydroxy-5-methyl-4-isoxazolepropionic acid receptor (AMPAR). The task-irrelevant background noise *I*_AMPA,n_ is mediated by AMPAR. The task-relevant currents *I*_e_ encodes the stimuli to E neurons. Each neuron receives a total synaptic current as:


(2) $$I_{\text{syn}}\,\left(t\right)=I_{\text{NMDA}}+I_{\text{GABA}}+I_{\text{AMPA},\text{n}}+I_{\text{e}}$$

We assumed that I neurons did not receive external input; thus, *I*_e_ = 0 for I neurons. The currents mediated by AMPAR, NMDAR, and GABAR to neuron *i* are modeled as follows:

$$I_{i,\text{AMPA},\text{n}}=\left(V_{i}-V_{\text{E}}\right)\,g_{\text{AMPA},\text{n}}\,S_{\text{AMPA},\text{n}}$$

(3) $$I_{i,\text{NMDA}}=\left(V_{i}-V_{\text{E}}\right)\,\sum_{j}\frac{g_{j\,i,\text{NMDA}}\,S_{j,\text{NMDA}}}{1+\left[M\,g^{2+}\right]\,\left[\exp\left(-0.062\,V_{i}\right)/3.57\right]}$$

$$I_{i,\text{GABA}}=\left(V_{i}-V_{\text{I}}\right)\,\sum_{j}g_{j\,i,\text{GABA}}\,S_{j,\text{GABA}}$$

[*Mg*^2 +^] = 1*mM*, *V*_*E*_ = 0*mV*, and *V*_*I*_ = −70*mV*; *g*_*AMPA,n*_ of *I*_*AMPA,n*_ are 2.48*nS* for E neuron and 1.9*nS* for I neuron. The AMPAR- and GABAR-related gating variables are determined by the presynaptic spike train {*t*_*k*_}:


(4) $$\frac{d\,S}{d\,t}=-\frac{S}{\tau_{S}}+\sum_{k}\delta \,\left(t-t_{k}\right)$$

where τ_*S*_ = 2;*ms* for AMPAR and τ_*S*_ = 10;*ms* for GABAR.

We introduced STP to recurrent connections mediated by NMDAR, as described in Gianluigi et al. (2008). The dynamics of the available resources *x*_*j*_(*t*) and utilization of resources *u*_*j*_(*t*) of presynaptic neuron *j* are described as follows:

$${\dot{x}}_{j}=\frac{1}{\tau_{x}}\,\left(1-x_{j}\right)-x_{j}\,u_{j}\,\sum_{k}\delta \,\left(t-t_{k}\right)$$

(5) $${\dot{u}}_{j}=\frac{1}{\tau_{u}}\,\left(U-u_{j}\right)+U\,\left(1-u_{j}\right)\,\sum_{k}\delta \,\left(t-t_{k}\right)$$

where the parameter *U* ∈ (0,1] and τ_*u*_ modulate the level of facilitation. The parameter τ_*x*_ controls the depression. We set τ_*u*_ = 1,650;*ms* and τ_*x*_ = 250;*ms*. Then, the NMDAR-related gating variable can be modeled as second-order dynamics (Albert et al., 2000):

$$\frac{d\,y}{d\,t}=-\frac{y}{\tau_{y}}+u\,x\,\sum_{k}\delta \,\left(t-t_{k}\right)$$

(6) $$\frac{d\,S}{d\,t}=-\frac{S}{\tau_{S}}+\alpha_{S}\,y\,\left(1-s\right)$$

where τ_*S*_ = 100;*ms*, α_*S*_ = 1;*ms*^−1^, and τ_*y*_ = 2*ms*.

### Network architecture

**FIGURE 1:**
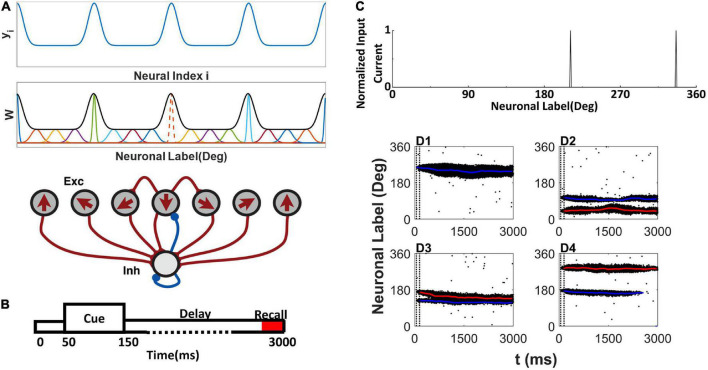
Network model structure, simulation protocol, and sample raster plot of simulation. **(A)** Model schemes. The network is composed of excitatory E neurons (Exc) and inhibitory I neurons (Inh) (bottom). E neurons are placed on a ring, labeled by their preferred directions (shown by arrows) representing the preferred color labeled by the angle in a color wheel. *y*_i_ is used to determine the $J_{i}^{+}$ (top). The EE connections between E neurons are structured as a Gaussian function of the difference between the preferred directions (middle). At middle, the orange dot line shows the sample normalized connection strength from neuron to other E neurons, and the black thick line shows the envelope curve revealing heterogeneous connection scheme. The connections onto and from the I neurons are uniform. **(B)** Simulation protocol. A cue array is presented to the network from 50 to 150 ms, followed by a delay period up to 3,000 ms. **(C)** Sample cue array of two random directions. **(D1–D4)** The raster plot of E neurons labeled with their preferred direction during simulation. The blue line shows the decoding angle θ_d,1_, and the red line shows the decoding angle θ_d,2_. The two vertical dash black lines indicate the cue period. **(D1)** There is one activity bump when presenting one color. **(D2–D4)** Three conditions of activity bumps when presenting two colors.

The network contains 512 E and 128 I neurons. Following the tradition of the ring model (Albert et al., 2000), each E neuron is placed on a circle according to its preferred color, indicated as θ_*i*_(0 < θ_*i*_≤2π) on the color wheel (Panichello et al., 2019). The arrow in each E neuron indicates the preferred color in Figure 1A. We assumed that all connections from or onto I neurons are uniform, and we set $g_{j\,i,\text{NMDA}}^{\text{EI}}=G_{\text{NMDA}}^{\text{EI}}$, $g_{j\,i,\text{GABA}}^{\text{IE}}=G_{\text{GABA}}^{\text{IE}}$, and $g_{j\,i,\text{GABA}}^{\text{II}}=G_{\text{GABA}}^{\text{II}}$. The synaptic conductance between E neurons obeys $g_{j\,i,\text{NMDA}}^{\text{EE}}=G_{\text{NMDA}}^{\text{EE}}\,W\,\left(\Delta \,\theta_{j,i}\right)$, Δθ_*j*,*i*_ = |θ_*j*_−θ_*i*_|| if |θ_*j*_−θ_*i*_| < π, otherwise, Δθ_*j*,*i*_ = |θ_*j*_−θ_*i*_|−π, *W*(Δθ_*j*,*i*_) is the footprint of the connection between E neurons shown at the top of Figure 1A:


(7) $$W\,\left(\Delta \,\theta_{j,i}\right)=J_{i}^{-}+\left(J_{i}^{+}-J_{i}^{-}\right)\,\text{ exp}\left[\frac{-{\left(\Delta \,\theta_{j,i}\right)}^{2}}{2\,\sigma_{i}^{2}}\right]$$

where σ_i_ reflects the effective cross-interaction range of E neuron i. By normalizing footprint $\frac{1}{2\,\pi }\,\int_{0}^{2\,\pi }W\,\left(\Delta \,\theta_{j,i}\right)\,d\,\theta_{\text{j}}=1$, we obtain $J_{\text{i}}^{-}=\frac{2\,\pi -\sqrt{2\,\pi }\,\sigma_{\text{i}}\,J_{\text{i}}^{+}}{2\,\pi -\sqrt{2\,\pi }\,\sigma_{i}}$. To explain the experimental observation in Panichello et al. (2019), we specifically tailored the connections between neurons to a heterogenous connectivity. We let $J_{\text{i}}^{+}=\bar{J}\,N\,y_{\text{i}}/\left(\sum_{j=1}^{N}y_{\text{j}}\right)$, where the intermediate variable $y_{\text{i}}=1-\frac{1}{b}\,\left[1-\frac{1}{\sqrt{2\,\pi }}\,\exp\left(\frac{{\left(i-\frac{N}{8}-\left[\frac{4\,i}{N}\right]\,\frac{N}{4}\right)}^{2}}{S_{N}^{2}}\right)\right]$ is a periodic function of neuron labels (preferring direction) and shown in the top panel of Figure 1A. The parameter b controls the heterogeneity of $J_{\text{i}}^{+}$. When *b* → + ∞, the connectivity is homogeneous. $\left[\frac{4\,i}{\text{N}}\right]$ denotes the floor of $\frac{4\,i}{N}$. *S*_N_ is the scale parameter related to neuron number *N*. We set *S*_N_ = 10 for *N=512*. $\bar{J}$ is the maximum value of $J_{\text{i}}^{+}$ and $J_{\text{i}}^{-}=\bar{J}+\frac{1-\bar{J}}{1-\sqrt{2\,\pi }\,\bar{\sigma }\,\text{erf}\,\left(\frac{1}{2\,\sqrt{2}\,\bar{\sigma }}\right)}$, where erf (*x*) is a Gaussian error function, and parameter $\bar{\sigma }$ determines the gap between $J_{\text{i}}^{-}$ and $J_{\text{i}}^{+}$. And σ_i_ of each neuron i can be determined by $J_{\text{i}}^{-}=\frac{2\,\pi -\sqrt{2\,\pi }\,\sigma_{\text{i}}\,J_{\text{i}}^{+}}{2\,\pi -\sqrt{2\,\pi }\,\sigma_{\text{i}}}$. The overall heterogenous connectivity is shown in middle panel of Figure 1A.

### Simulation protocol

The simulation protocol is illustrated in Figure 1B. The stimuli are presented to the network during the cue period from *50* to 150*ms*. The E neuron θ_*i*_ receives input from the *α-*th stimulus, which is located at θ_α_ on the color wheel, and the total input to neuron θ_*i*_ is as follows:


(8) $$I_{\text{e}}\,\left(\theta_{i}\right)=\sum_{\alpha }^{n}\frac{I_{0}}{\sqrt{2\,\pi }\,\sigma_{s}}\,\exp\left[-\frac{{\left(\theta_{i}-\theta_{\alpha }\right)}^{2}}{2\,\sigma_{s}^{2}}\right]$$

where *n* is the number of stimuli, σ_s_ is the effective range of the external input. We set σ_*s*_ = 2°. Two random stimuli cue array are shown in Figure 1C.

In addition to the external input, each neuron receives a background Poisson spike train {*t*_*k*_} with a mean arrival rate of 1 kHz, which is transferred into background noisy currents through the AMPAR.

We used the custom code in MATLAB to simulate our model using the RK2 method. We varied the parameters *U*, *b*, *j*, and $\bar{\sigma }$ to explore the dynamics of the network. For a single set of parameters, we made statistics on the data of 4,000 trials, giving each set of parameters. We found that the parameters set *U* = 0.8, *b* = 1.2, $\bar{J}=4.9$, and $\bar{\sigma }=4.0{}^{{}^{\circ}}$ can reproduce the experimental observations in Panichello et al. (2019) and we presented the results based on these parameters value without specific statement throughout the manuscript.

### Decoding method

We followed the previous subpopulation vector method to decode the neural activity (Wei et al., 2012). We divided the neurons into different populations according to the stimulus and calculated the population vector of the subpopulation of the α-th stimulus as the memory trace of the stimulus: θ_*d*, α_ = *arg* [∑_*j*∈*N*α_ *r*_*j*_ (*t*) exp (iθ_j_)], where *r*_j_(*t*) is the firing rate of E neuron j that prefers θ_j_, *N*_α_ is a subpopulation of E neurons that are related to the α-th stimulus. The number of subpopulations equals to the number of input colors. We calculated the firing rate of each neuron at each time. One neuron belongs to one closet activity bump if its firing rate is higher than baseline activity (2 Hz). Then we can segment the whole population of E neurons into subpopulations.

## Results

### Dynamic representation of memorized information

The activity pattern of the network was consistent with previous studies (Albert et al., 2000; Christos and Wang, 2004; Wei et al., 2012). During the cue period, the stimulus evoked spikes of neurons whose preferred color was close to that of the stimulus. The responses of the neurons formed a localized activity bump in the raster plots (Figures 1D1–D4). The activity bump persisted throughout the delay period given one stimulus (Figure 1D1). There were three different consequences for the two stimuli. Each activity bump persisted throughout the delay period (Figure 1D2); two activity bumps may merge into one activity bump (Figure 1D3). One of the activity bumps persisted, but the other activity bump faded during the delay period (Figure 1D4). Once the activity bump fades away, the network forgets the stimulus information, and the network can only randomly report a color.

The localized activity bump can be decoded as a memory trace using the subpopulation vector decoding method (described in the Section “Materials and methods”). The decoded memory traces are shown as red or blue lines in the raster plot (Figures 1D1–D4). The memory trace can deviate from the original input color, leading to the clustering report and report error.

### Clustering report and report error distribution

In our simulation, we presented one or two colors sampled from 0*to* 360° with equal probability, which is consistent with what Panichello et al. (2019) have performed in their experiment. If the activity bump persists throughout the delay period, we use the memory trace during the recall period θ_*d*, α_ (*t*_end_) as the report of the network. If the activity bump fades, we choose a random value of the color as the network report. The report error is the difference between the input stimulus and network report: θ_*d*, α_ (*t*_end_) − θ_α_, which is then rescaled to [-180,° 180]°.

**FIGURE 2:**
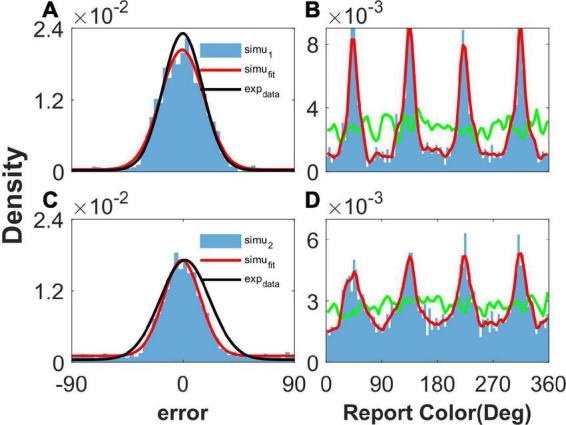
The distribution of report error and report in the simulation. Panels **(A,B)** show the results given one color, and panels **(C,D)** show the results given two concurrent stimuli. **(A,C)** The distribution of report error. The blue histogram shows the distribution of report error of the network, and the red line is the Gaussian fit curve of the histogram. The black line is the Gaussian fit curve of distribution of report error in the experiment (from Panichello et al., 2019). **(B,D)** The distribution of report. The blue histogram shows the distribution of report of the network, and the red line is the smooth line of the histogram. The green line indicates the distribution of input color. The parameters are set as follows: *U* = 0.8, *b* = 1.2, $\bar{J}=4.9$, and $\bar{\sigma }=4.0{}^{{}^{\circ}}$.

After the simulation, we performed statistical analysis on reports and report errors to obtain the probability density distribution of both, as performed in Panichello et al. (2019), which are shown in Figure 2.

When presenting one color, we statistically obtained the distribution of report error in the simulation (Figure 2A), as well as the distribution of report in the simulation (Figure 2B). In Figure 2A, the distribution of report error is shown as a blue histogram fitted by a Gaussian curve (red line). The black line is the Gaussian fit of report error in the experiment by Panichello et al. (2019). The red line almost overlaps with the black line, indicating that our model reproduces the distribution of report error. In Figure 2B, the green line shows the distribution of input color, the blue histogram indicates that the report of the network is clustering, and the smooth red line of the histogram indicates clusters clearly.

We also presented two colors in the simulation. The difference between the two stimuli is larger than 25°, which is similar to the stimuli presented to the participants in the experiment (Panichello et al., 2019). The simulated distribution of report error is close to that of the experiment (Figure 2C). The report of the network is clustering, given two stimuli (Figure 2D). In brief, our network model reproduces the typical phenomena that the report of a stimulus sampled from a uniform distribution is clustering and the report error follows a Gaussian distribution.

### Effects of heterogeneous connection on memory reports

**FIGURE 3:**
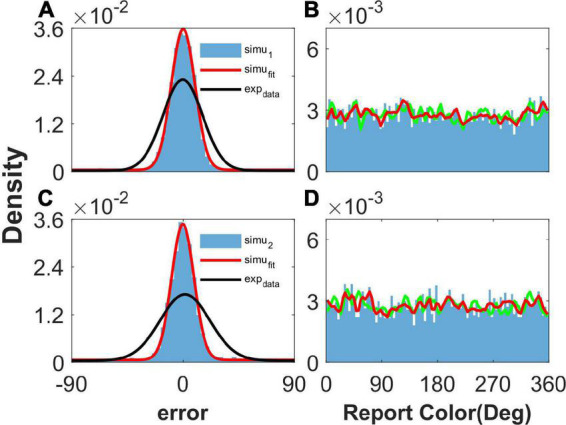
The distribution of report and report error with short-term plasticity (STP) but without heterogenous connectivity. Panels **(A,B)** show the results given one stimulus, and panels **(C,D)** show the results given two stimuli. **(A,C)** The distribution of report error. The blue histogram shows the distribution of report error of the network, and the red line is the Gaussian fit curve of the histogram. The black line is the Gaussian fit curve of distribution of report error in the experiment (from Panichello et al., 2019). **(B,D)** The distribution of report. The blue histogram shows the distribution of report of the network, and the red line shows the smooth line of the histogram. The green line indicates the distribution of input color. The parameters are set as follows: *U* = 0.8, *b* = 10,000, $\bar{J}=4.9$, and $\bar{\sigma }=4.0{}^{{}^{\circ}}$.

As shown in Figure 2, our model can reproduce the clustering report and the Gaussian-like distribution of report error observed in the experiment (Panichello et al., 2019). The discrete attractors in the WM model are thought to be the mechanism leading to clustering report (Panichello et al., 2019). In our model, we introduced heterogeneity into the connectivity of the original ring model (Albert et al., 2000). The hallmark of the original ring model is its continuous attractor owing to the translation invariance of connectivity. The introduction of heterogeneity breaks up the translation invariance, and a continuous attractor does not exist, leading to discrete-like attractors in the network. Thus, when we set *b* = 10,000 to make the heterogeneity of the connections sufficiently small to be negligible, the continuous attractor returns, and the report of the network is not clustering (Figures 3B, D). In particular, the distribution of report (red line) almost overlaps with the distribution of the stimulus (green line), indicating that heterogeneity is necessary for the clustering report. At the same time, the report error follows a Gaussian distribution, but the deviation of the report error is much smaller than that from the experiment (Figures 3A, C).

### Effects of STP on memory reports

**FIGURE 4:**
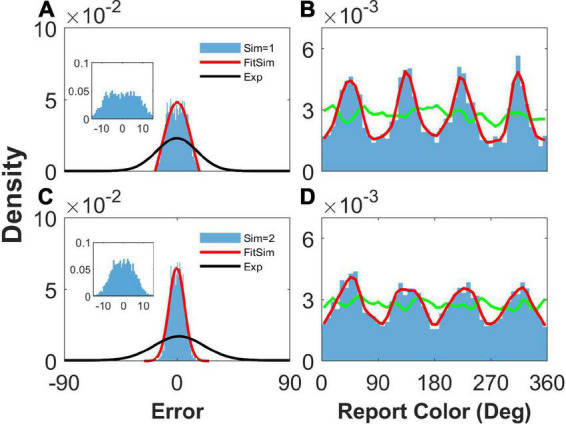
The distribution of report error and report with heterogenous connectivity but without short-term plasticity (STP). Panels **(A,B)** show the distribution when presenting one stimulus, and panels **(C,D)** show that when presenting two stimuli. **(A,C)** The distribution of report error. The blue histogram shows the distribution of report error in the simulation, and the red line is the Gaussian fit curve of the histogram but the negative part is truncated. The black line is the Gaussian fit curve of distribution of report error in the experiment (from Panichello et al., 2019). **(B,D)** The distribution of report. The blue histogram shows the distribution of report in the simulation, and the red line shows the smooth line of the histogram. The green line indicates the distribution of input color. The parameters are set as follows: *b* = 1.2, $\bar{J}=4.9$, and $\bar{\sigma }=4.0{}^{{}^{\circ}}$. The SSE (the sum of squares due to error) of Gaussian fit in panels **(A,C)** is 0.0013/0.00003.

We then removed the STP mechanism from Equation 6 by replacing *ux* with constant *1* to explore the effects of STP on the network report. We set *b* = 1.2, $\bar{J}=4.9$, and $\bar{\sigma }=4.0{}^{{}^{\circ}}$ to ensure that the heterogeneous connection was the same as that in Figure 2. We found that the network reports were still clustering (Figures 4B, D), indicating that STP has a slight effect on the clustering report. However, the STP has a significant effect on the distribution of report error. We can see that the histogram of the report error significantly deviated from the experimental observation. First, the distribution of the simulated report error has a plateau and deviates from the Gaussian distribution (Figures 4A, C). The plateau of the simulated report error can be clearly seen in the inset of the Figures 4A, C. Second, the width of the histogram of the report error given two stimuli is smaller than that given one stimulus. However, experimental observations indicate that the deviation of the report error increases with the number of stimuli. Third, the width of the distribution of the simulated report error is much smaller than that of experimental observation, indicating that the drift of the activity bump in the network without STP is not enough. Thus, the distribution of report error given by the network without the STP is inconsistent with the experimental observation.

### Effects of STP on the spatiotemporal pattern of the membrane potential of our model

Because of the presence of heterogeneous connections, it is difficult to reveal the dynamic mechanism of STP in an analytical manner, as in Alan et al. (2012) and Alexander et al. (2019). However, we were able to analyze the dynamic effects of STP on the activity bump by analyzing the change in the spatiotemporal pattern of the membrane potential of E neurons in the network with or without STP when presenting one stimulus.

**FIGURE 5:**
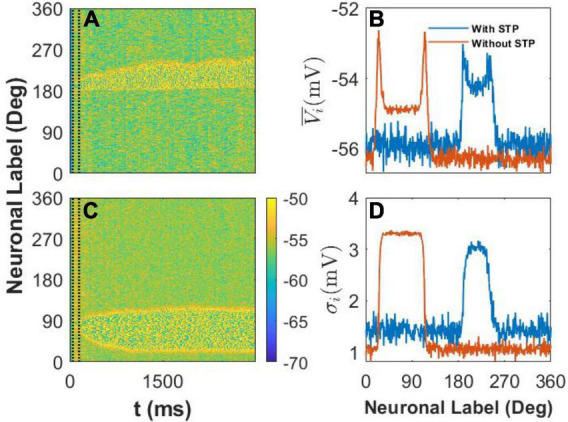
The spatiotemporal pattern and statistics of membrane potential, *Vi*. **(A)** The spatiotemporal pattern of the model with short-term plasticity (STP). The color bar shows that *Vi* ∈ [−70 mV, −50 mV], and two vertical dash black lines indicate the cue period. The stimulus color θ_α = 1_ = 190°. **(B)** The spatiotemporal pattern of the model without the STP. The stimulus color θ_α = 1_ = 85°. **(C)** The mean membrane potential ${\bar{V}}_{i}$ of every E neuron during the last 1,000ms. The blue and red lines indicate the statistical results with the effect of STP or without it, respectively. **(D)** The standard deviation σ_*i*_ of E neurons during the last 1,000ms. The blue and red lines indicate the same condition in panel **(C)**.

We recorded the membrane potential *V*_*i*_ of every E neuron *i* at 1-ms intervals and then drew the spatiotemporal pattern, that is, the heat map of *V*_*i*_ in Figures 5A–D. We also calculated the mean value ${\bar{V}}_{i}$ and standard deviation σ_*i*_ of *V*_*i*_ during the last 1,000*ms* of the simulation to clarify the difference between the two spatiotemporal patterns. First, in our model, we set *U* = 0.8, and the STP is depression; thus, the STP plays an inhibitory role in the activity of E neurons. As a result, the width of the activity bump between the slender yellow area in Figure 5A with STP was narrower than that in Figure 5B without STP. Second, the membrane potential of neurons outside the activity bump exhibited a slow subthreshold oscillation owing to the effect of the STP (Figure 5A). However, the membrane potential of the E neurons outside the activity bump showed a uniform noisy pattern with smaller fluctuations (Figure 5B).

We further calculated the mean and standard deviation of the membrane potential of each E neuron during the last 1,000*ms* of the delay period. We found that STP increased the mean membrane potential ${\bar{V}}_{i}$ of E neurons outside the activity bump and also increased the ${\bar{V}}_{i}$ of E neurons inside the activity bump (Figure 5C). At the same time, STP increased the standard deviation σ_*i*_ of E neurons outside the activity bump but decreased the standard deviation σ_*i*_ of the membrane potential of neurons inside the activity bump (Figure 5D). STP can decrease the stability and increase the drift of the activity bump by increasing the membrane potential and enlarging the fluctuation of the membrane potential of E neurons outside the activity bump. As a result, the STP can enhance the drift of the activity bump elicited by the stimulus to discrete attractors determined by heterogeneous connectivity.

### The spatiotemporal pattern of the variables of STP

**FIGURE 6:**
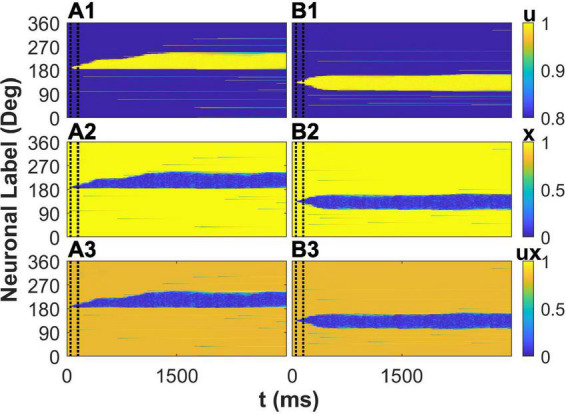
The spatiotemporal pattern of the variables of short-term plasticity (STP). The cue stimulus is θ_α = 1_ = 190° for panels **(A1–A3)** and θ_α = 1_ = 135° for panels **(B1–B3)**, respectively. The spatiotemporal pattern of utilization of resource u (top), available resource x (middle), and the overall plasticity ux (bottom).

Besides the effects of STP on membrane potential of E neurons in the network, we further investigated the spatiotemporal pattern of the variables of STP to reveal the mechanism of the enhancement of the drift of the activity bump by STP. We first explored the situation that a cue (190°) is far from the peak of the connectivity. The cue elicits the activity of neurons preferred 190° during cue period. The synaptic strength from these activated neurons to neuron whose preferred color is larger than 190° are stronger than to neuron whose preferred color is smaller than 190° due to that 190° is closer to one peak of connectivity (225°) than to the other peak (135°). The asymmetric connections to the initially activated neurons lead to asymmetric spatiotemporal pattern of variables of STP: utilization of resource *u*, available resource *x*,and the overall STP *ux*, respectively (Figures 6A1–A3). This depressed plasticity leads to weaker synaptic outputs from neuron whose synapse was depressed as shown in blue area in Figure 6A3. Therefore, the neurons closer to the peak of connectivity (225°) received more asymmetric synaptic inputs, driving the elicited activity bump far from the initial cue location toward the peak of the connectivity and leading to a larger deviation of report error. Due to the mobility of activity bump induced by STP, the plateau of distribution of the report error (Figure 4) was eliminated. We then investigated the situation that a cue (135°) is close to the peak of the connectivity. The spatiotemporal pattern of STP variables is almost symmetry (Figures 6B1–B3) because the initially activated neurons during the cue period have almost symmetric influence on their projected neurons. As the result, the activity bump will stay near the peak of the connectivity with small fluctuation. In summary, consistent with the analysis on membrane potential of E neurons, STP causes a larger deviation by increase the mobility of the activity bump far from the peak of activity bump, leading to a larger deviation of the distribution of the report error and eliminating the plateau of the distribution of the report error by the network with heterogenous connectivity but without STP.

## Conclusion and discussion

In this study, we constructed a spiking neuron network model with two main mechanisms: heterogeneous connection and STP. The model can reproduce the distribution of the report error and report observed in the experiment (Panichello et al., 2019). We found that the heterogeneity connection between E neurons plays a key role in clustering report and the STP enhances the drift of the activity bump to modulate the report error.

**FIGURE 7:**
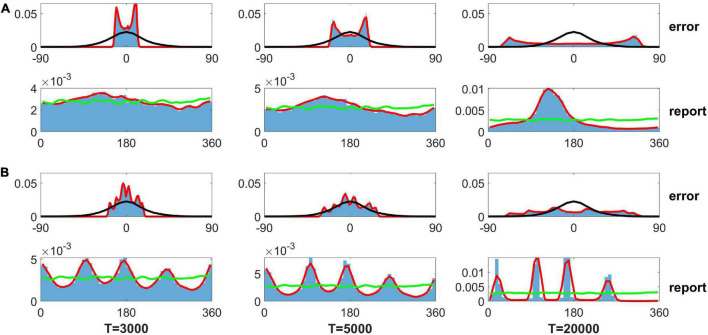
Distribution of the report error and report from the stochastic differential equation (SDE) in Panichello et al. (2019). Panel **(A)** shows the results using parameters fitted by experimental data in literature (Panichello et al., 2019), and panel **(B)** shows the results based on the same parameters, except for heterogeneity controlling parameters. The top rows of panels **(A,B)** show the distribution of report error. The bottom rows of panels **(A,B)** show the distribution of report. The blue histograms denote the distribution of report error in the simulation. The red lines are the smooth curve of the histogram. The black lines denote the Gaussian fit of distribution of report error in the experiment. The green lines indicate the distribution of input color. The delay period times are 3,000, 5,000, and 20,000 s for the left, middle, and right column, respectively. The parameters in panel **(A)** are set as follows: {*w*_*j*_} {6.26, 8.22, 9.75, 7.89, 7.48, 10.28, 7.23, 7.75, 9.62, 7.74, 6.48, 11.27} × 10^−2^. The parameters in panel **(B)** are set as follows: {*w*_*j*_} {6.26, 8.22, 59.75, 7.89, 7.48, 60.28, 7.23, 7.75, 59.62, 7.74, 6.48, 61.27} × 10^−2^.

As mentioned in the Section “Introduction,” Panichello et al. (2019) used the SDE, *d*θ = β_*L*_*G*(θ)*dt* + σ_*L*_*dW*, to describe the evolution of the memory trace. The β_*L*_ sets the gain of the drift, and the $\sigma_{L}^{2}$ is the variance of the Gaussian white noise. *L* is the number of input color. $G\left(\theta \right)=\sum_{j}^{12}w_{j}\frac{d}{d\,\theta }\phi (\frac{2\,\pi }{12}j,\frac{2\,\pi }{12}$, where the ϕ is a von Mises distribution. They assumed that the evolution of the memory trace is controlled by discrete attractors (drift role) and Gaussian white noise (diffusion role). They further determined the distribution of memory traces using FPE and fitted their experimental data. Although this distribution obtained from the FPE fits the experimental data well, it is difficult to obtain similar results by direct simulation of the SDE. Here, we solve the SDE using the modified Euler–Heun method (Christopher and Nie, 2017). We first used the parameters in Panichello et al. (2019): β1 = 0.0917, σ_1_ = 3.637×10^−4^, and {*w*_*j*_} are shown in the caption of Figure 7. When we get the evolved angle θ, following the fit of FPE by Panichello et al. (2019), we use the guess rate λ to determine if the report angle θ is replaced by a random guess angle. Because the input contains only one angle (Load 1 condition in experiment), we set the swap rate α as zero. We found that reports by SDE can be clustered but obviously deviate from the experimental data given different delay periods of 3,000, 5,000, and 20,000 s for the left, middle, and right panels of the bottom row in Figure 7A, respectively. The distribution of report error exhibits a bimodal distribution but not a Gaussian distribution (top row in Figure 7A). We further changed the parameter {*w*_*j*_} to enhance the effects of the heterogeneity in SDE but did not change β_*i*_ and ${\bar{\sigma }}_{i}$ of SDE. For the new set of values {*w*}, we added a fixed value at the four peaks of original values {*w*_*j*_} (shown in the caption of Figure 7) to make a stronger heterogeneity of von Mises distribution used in Panichello’s model. Although the distributions of report were clustered (bottom row in Figure 7B), the distribution was inconsistent with the experimental observations. The distribution of report error exhibits a multimodal but not a Gaussian distribution. In brief, the results from directly solving the SDE indicate that memory traces driven by discrete-like attractors and Gaussian white noise cannot explain the clustering report and Gaussian distribution of report error. In contrast, our model with heterogeneous connectivity and STP can account for experimental observations. For our model, several points are worthy of noting.

First, we directly introduced heterogeneity to the connectivity in our model. This heterogeneity in connectivity may result from synaptic plasticity due to repetitive exposure to external stimuli during development (Li et al., 2022). Another possible mechanism is the synaptic plasticity such as Hebbian learning. Considering that Hebbian learning rule drives the synaptic weight approach to the direction of the principal component of external inputs, this mechanism could be used to explain the observation that the experience is likely to be used for getting the wrong guess of color to modify the distribution (Panichello et al., 2019). Thus, the heterogeneity in the network is still not clear.

Second, in the original ring model, the connectivity is translation invariance, which implies that the network can elicit one activity bump located at any position and the activity bump just diffuses along with time owing to the noise (Albert et al., 2000). Heterogeneity breaks up the translation invariance and impairs the stability of the activity bump (Song et al., 2003), which implies that the heterogeneity of connectivity prefers the drift of the activity bump. Moreover, the connection between neurons located at two peaks of connectivity is asymmetry in our model, which causes the activity bump drift toward the peak of the connectivity (Huang et al., 2015). Besides the heterogenous connectivity, the random connectivity can also break up the translation invariance and generate attractors in the network (Hansel and Mato, 2013). However, the random connectivity will lead to random location of attractors, which is inconsistent with the experimental observations. One recent research on the color memorizing and retrieval showed that individual subjects partitioned the continuum hues into discrete categories and exhibited focal colors in their own mnemonic strategy (da Fonseca et al., 2019), which says that the connectivity should not be random.

Third, the heterogeneity of connectivity in our model leads to discrete-like attractors, and the activity bump approaches the attractors, leading to clustering report. However, the drift driven by the asymmetric connection between neurons causes the report error to deviate from a Gaussian-like distribution (Figures 4, 7). Therefore, we had to introduce a new mechanism to modulate the drift of the activity bump. Previous research has shown that the STP may induce a traveling wave in the ring model (Alan et al., 2012) or control the stability of the WM (Itskov et al., 2011; Alexander et al., 2019). Thus, we used the STP to modulate the drift of the activity bump and reproduce typical phenomena in the experiment. We chose a larger value of the baseline of the utilization of resource (*U* = 0.8). The larger U implies that the larger portion of available ready transmitter can be released into cleft. Thus, the large U favors the depression of the synaptic efficacy. The experiments showed the release probability of neural transmitter varies from 0.1 to 0.9 in different synaptic connections and different species (Branco and Staras, 2009). The smaller U makes the bump more stable which is inconsistent with the experimental results.

**FIGURE 8:**
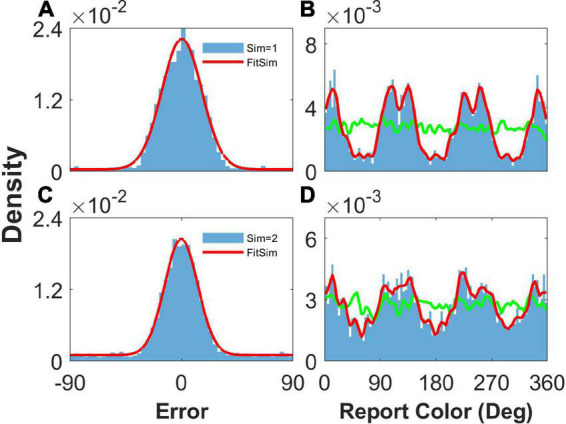
Result of heterogeneous connection with three peaks. Panels **(A,B)** show the distribution when presenting one stimulus, and panels **(C,D)** show that when presenting two stimuli. **(A,C)** The distribution of report error. The blue histogram shows the distribution of report error in the simulation, and the red line is the Gaussian fit curve of the histogram. The black line is the Gaussian fit curve of distribution of report error in the experiment. **(B,D)** The distribution of report. The blue histogram shows the distribution of report in the simulation, and the red line shows the smooth line of the histogram. The green line indicates the distribution of input color. The parameters are set as follows: *U* = 0.8, *b* = 1.2, $\bar{J}=4.9$, and $\bar{\sigma }=4.0$°.

Fourth, our model can reproduce not only the clustering report with four clusters, which is consistent with the report distribution of humans in the experiment (Panichello et al., 2019), but also the clustering report with three clusters by changing the structure of the heterogeneous connection. We only adjusted the parameters controlling the number of peaks and simulated the model. We can see that the report was clustered with one stimulus (Figure 8B) or two stimuli (Figure 8D). The report error followed a Gaussian distribution (Figure 8). In summary, our model can account for the clustering report and the Gaussian distribution of report error.

## Data availability statement

The original contributions presented in this study are included in this article/supplementary material, further inquiries can be directed to the corresponding author.

## Author contributions

D-HW and MZ conceptualized the research. YD, TL, and LL carried out the simulation. D-HW and LL wrote the manuscript. All authors contributed to the article and approved the submitted version.
